# Synergistic Effects of 3D ECM and Chemogradients on Neurite Outgrowth and Guidance: A Simple Modeling and Microfluidic Framework

**DOI:** 10.1371/journal.pone.0099640

**Published:** 2014-06-10

**Authors:** Parthasarathy Srinivasan, Ioannis K. Zervantonakis, Chandrasekhar R. Kothapalli

**Affiliations:** 1 Department of Mathematics, Cleveland State University, Cleveland, Ohio, United States of America; 2 Department of Cell Biology, Harvard Medical School, Boston, Massachusetts, United States of America; 3 Department of Chemical and Biomedical Engineering, Cleveland State University, Cleveland, Ohio, United States of America; Vanderbilt University Medical Center, United States of America

## Abstract

During nervous system development, numerous cues within the extracellular matrix microenvironment (ECM) guide the growing neurites along specific pathways to reach their intended targets. Neurite motility is controlled by extracellular signal sensing through the growth cone at the neurite tip, including chemoattractive and repulsive cues. However, it is difficult to regenerate and restore neurite tracts, lost or degraded due to an injury or disease, in the adult central nervous system. Thus, it is important to evaluate the dynamic interplay between ECM and the concentration gradients of these cues, which would elicit robust neuritogenesis. Such information is critical in understanding the processes involved in developmental biology, and in developing high-fidelity neurite regenerative strategies post-injury, and in drug discovery and targeted therapeutics for neurodegenerative conditions. Here, we quantitatively investigated this relationship using a combination of mathematical modeling and *in vitro* experiments, and determined the synergistic role of guidance cues and ECM on neurite outgrowth and turning. Using a biomimetic microfluidic system, we have shown that cortical neurite outgrowth and turning under chemogradients (IGF-1 or BDNF) within 3D scaffolds is highly regulated by the source concentration of the guidance cue and the physical characteristics of the scaffold. A mechanistic-driven partial differential equation model of neurite outgrowth has been proposed, which could also be used prospectively as a predictive tool. The parameters for the chemotaxis term in the model are determined from the experimental data using our microfluidic assay. Resulting model simulations demonstrate how neurite outgrowth was critically influenced by the experimental variables, which was further supported by experimental data on cell-surface-receptor expressions. The model results are in excellent agreement with the experimental findings. This integrated approach represents a framework for further elucidation of biological mechanisms underlying neuronal responses of specialized cell types, during various stages of development, and under healthy or diseased conditions.

## Introduction

Ever since Ramón y Cajal conjectured that chemotaxis might help guide neurites to their intended targets, numerous studies have sought to identify the ubiquitous role of various neurotrophic factors and their gradients on neurite outgrowth and targeting [1]–[3]. In the embryonic and postnatal CNS, a myriad of diffusible and surface-bound cues steer the growing neurites to facilitate specificity in neuronal connections and precision in neural circuit wiring [4]. The motile growth cones at the tip of growing neurites sense the spatio-temporal distribution of guidance cues within that extracellular microenvironment and navigate toward (attraction) or away (repulsion) from the gradient to reach their target [5]. Specifically, gradients of these cues induce a localized intracellular signaling cascade resulting in active polymerization and depolymerization of the cytoskeletal components (actin & microtubules) in growth cones, to initiate, direct, consolidate and stabilize the newly formed neurite extensions [6]–[8]. Interestingly, the same cues which regulate neurite guidance have also been implicated in initiating migration of neuronal cell bodies [9]. A thorough mechanistic understanding of the effect of biomolecular gradients on neurite dynamics within 3D microenvironments is not only relevant for developmental neuroscience but also critical to efforts toward stimulating directional neurite regrowth after an injury or disease.

Experimental studies conducted both *in vivo* and *in vitro* suggested that depending on the cell type and soluble molecule investigated, neurite outgrowth and guidance is greatly influenced by the molecular concentration (10^−13^–10^−3^ M), gradient steepness (0.01–10%) and direction (attractive vs. repulsive), and substrate topography (smooth vs. patterned, diffusive vs. surface-bound, etc.) [10]–[16]. Molecular cues could bind to the receptors on growth cone and soma membrane, activate effectors via receptor-cue complexation, to finally modulate actin dynamics [17]. Yet, much remains to be learned with regard to the dynamic interplay between physiological concentration ranges of gradients which elicit sharp neurite response, and the ECM microenvironment (composition, stiffness) which facilitates this process. Subtle variations in spatio-temporal concentrations, exposure time, intracellular and extracellular calcium levels, matrix heterogeneity and cell cycle stage could dramatically influence neurite response in complicated and unpredictable ways [17]. On the other hand, mounting evidence suggests that the growth cone guidance of neuronal subpopulations is synergistically regulated by multiple concurrent and at times contradictory cues, to result in events such as random extension and retraction [16], [18], [19]. *In vivo* studies are beneficial in identifying cues relevant to neural development, but it is difficult to control the microenvironment around the neurons and evaluate the role of the multitude of cues to which the growth cones are exposed. Despite recent advances in imaging and molecular biology, it is experimentally challenging to investigate and quantify the mechanisms by which molecular gradients regulate neurite dynamics using conventional culture techniques.

Several theoretical models and numerical simulations have been developed to understand axonal guidance, based on competing stochastic [20]–[25] (e.g., fractal growth, probabilistic distributions, obstacle avoidance) or deterministic [17], [26], [27] (e.g., activity of cell adhesion molecules, response to chemotrophic agents) factors that influence growth cone dynamics. A simple *persistent random walk* model often used for studying cell migration was employed to describe stochastic elements of growth cone migration [28]–[31]. Other important theoretical and computational models of axonal guidance were based on receptor distribution, gradient detection, cytoskeletal forces and lamellar bias [26], [32]–[36]. These models rightly assume that growth cones sense chemogradients spatially and temporally to activate corresponding surface receptors; however mechanistic details and prognostic assessment of how it affects neurite outgrowth is lacking. We hypothesize that numerical simulations using a mathematical model, coupled with parameter estimation from accurately-controlled experiments, might help in the identification of the mechanistic role of chemogradients on growth cone movement, and help to better interpret the experimental data. Such experimental investigations of gradient-induced growth cone migration and neurite outgrowth would clearly benefit from a biomimetic 3D culture system in which neurons are seeded within a physiological 3D matrix and subjected to quantifiable and highly-reproducible chemogradients.

**Figure 1:**
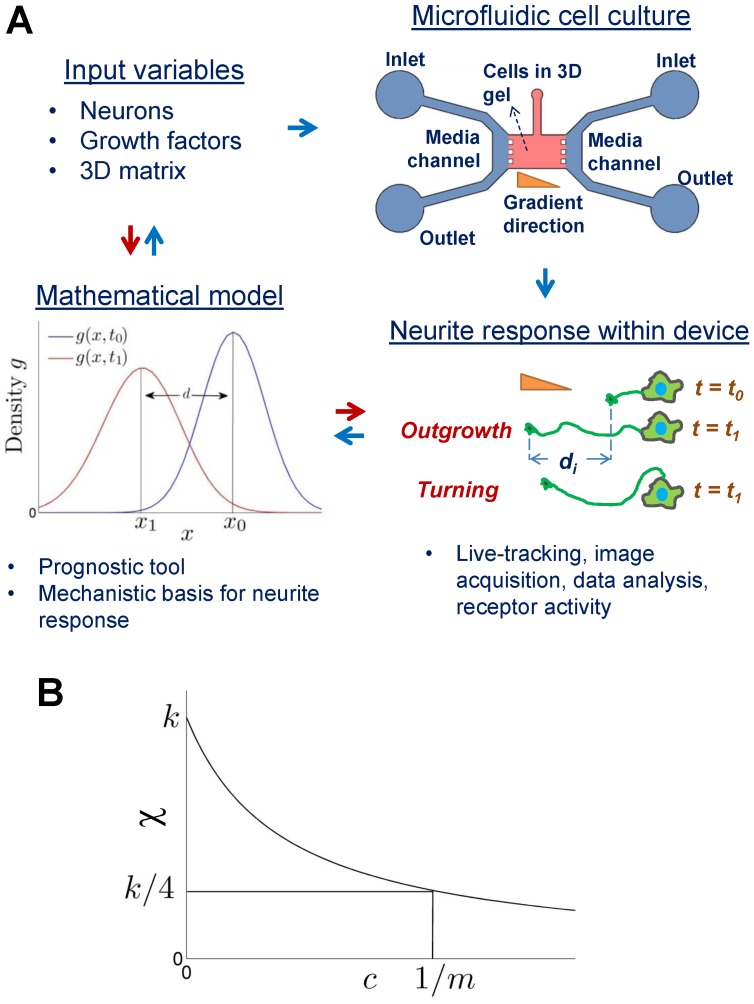
Integration of microfluidic platform and mathematical modeling. (A) Schematic outline of this study. Input variables for experimental determination of their synergistic roles in neurite outgrowth within a microfluidic device. The device permits neuronal culture within 3D biomimetic environment, and allows manipulation of variables. Neurite response could be observed *in situ* at fixed time-points or by live-tracking. Mathematical modeling provides a mechanistic basis and prediction of neurite outgrowth. If the distance moved by a growth cone of *i* ^th^ neuron due to chemotaxis in time *t_i_−t_0_* is given by *d_i_ =  |x_i_−x_0_|*, then the average distance moved by all cultured neurons will be *d = <d_i_>*. Here, *x_i_* is value of *x* at which max *g(x, t_i_)* is attained, where *g(x, t_i_)* is the growth cone density at time *t_i_*. (B) Graph of the chemotaxis term 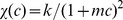, where *k = χ(0)* and *χ(1/m)  = k/4*.

In this work, our objective was to develop a simple mathematical model aimed at quantifying neurite outgrowth, i.e., growth cone migration from soma, as a function of the chemogradient concentration and the diffusion coefficient of biomolecule within that matrix, and propose a mechanism for chemotaxis that requires further investigation to elucidate the biochemical pathways involved (Fig. 1A and Graphical Abstract S1). Our mathematical framework builds on the chemotaxis model originally developed by Patlack, and later by Keller & Segel (K-S model) [37], [38]. Results from our model suggest that the concentration of the cue, its biomolecular diffusion coefficient and the domain length critically influences neurite outgrowth. To complement our model, we developed a microfluidic cell culture system to investigate and quantify the effect of various chemogradients on cortical neurite outgrowth within 3D ECM scaffolds. We experimentally demonstrate that in addition to the concentration gradient of diffusing cue, neurite outgrowth and turning within were also modulated by the composition and stiffness of 3D ECM scaffold (Fig. 1A). The model parameters were estimated using experiments under certain conditions. Then the model was used to predict the neurite outgrowth under different experimental conditions than those used for estimating parameters, and the model predictions agreed with the experimental validation. Furthermore, we provide evidence that such neuritogenesis under chemogradients was mediated by respective growth factor receptors on the cell surface. Taken together, our model and experimental methodologies present a reliable, comprehensive tool to develop and quantify strategies for determining and predicting the effects of physiologically-relevant cues to neurite outgrowth and targeting.

## Materials and Methods

### Microfluidic device fabrication

The device was designed using SolidWorks (*device dimensions in Fig. S1*) and fabricated at Stanford Microfluidics Foundry using standard photolithography and soft-lithography techniques [39]. The device has a central gel chamber (5 mm long, 2.7 mm wide, 150 µm height) flanked by media channels on either side, and a separate gel channel (3.5 mm long, 375 µm wide) to inject hydrogels. The media channels are connected to individual inlet and outlet ports at the ends. As we detailed earlier [40], [41], microfluidic chips were fabricated using poly-dimethylsiloxane (PDMS; Dow Corning) and crosslinking agent (10∶1 ratio), degassed, poured on the SU-8 wafer, and cured for 2 h at 80 °C. Individual devices were cut out (each 1.25″ diameter), media and gel filling ports cored using biopsy punches (Harris uni-core), cleaned and boiled in water for 30 min to remove uncrosslinked elastomer, and dried. The devices and glass cover slips were air plasma-treated for 1 min (Electro-technic Products Inc., BD 10-A), bond together to complete the device fabrication and stored.

### Gradient simulation and visualization

Gradient studies within the microfluidic device were performed by filling the central gel chamber with collagen hydrogel (rat-tail derived, type I, BD Biosciences), reconstituted at 2 mg/mL concentration using 10× PBS and 1 N NaOH, and the pH adjusted to ∼7.2. After collagen polymerization in a humidified incubator (37 °C, 30 min), PBS was added to the right channel and a dilute solution of FITC-Dextran (10 nM, 20 kDa, Molecular Probes) added to the left channel, to effectively create a gradient across the 3D collagen gel from left to right channels. Using an Axiovert A1 Zeiss epi-fluorescence microscope with an attached Hamamatsu camera, a series of florescent images were acquired at frequent intervals over 48 h, and images processed using a custom-written MATLAB code to quantify time-dependent changes in florescence intensity across the gel. The concentration gradients under different conditions were also quantified and analyzed parametrically using a diffusion-convection-reaction finite element model in COMSOL (Burlington, MA) package. The constant concentration source conditions at the inlet, and sink conditions at the outlet channels and at the gel filling port, were defined at the numerical model boundaries. The diffusion coefficients for the 17 kDa and 27 kDa molecular weight in 2 mg/mL collagen were calculated to be 8×10^−12^ m^2^/s and 5×10^−12^ m^2^/s, respectively [42], [43]. The numerical grid for performing the simulations consisted of ∼800,000 finite elements. Data obtained from COMSOL was analyzed and plotted using custom-written code in MATLAB.

### Neuron culture within devices

Cortical neurons were chosen in this study as they are easy to isolate from murine brain, and one of the widely studied neuronal populations. Although IGF-1 and BDNF have been widely explored in the context of their roles in developmental biology and post-injury in CNS, their optimal concentrations and gradients which promote the final effects have not been completely elucidated. E-18 rat primary cortical tissue (freshly isolated, not frozen) was obtained from Neuromics Inc. (Minneapolis, MN), and cortical neurons isolated as per recommended protocols using enzymatic solution containing papain but not B27. Three different types of scaffolds were tested: collagen-1 was reconstituted at 2 mg/mL and 1 mg/mL concentrations as described earlier, while growth factor-reduced matrigel (BD Biosciences) was used as-received. Cells were mixed in gels at 1∶10 ratio (∼500 cells per device) and filled within the designated gel chamber in microfluidic device through the gel-filling port. Devices were incubated in humidified incubator (37 °C, 30 min) to ensure gel polymerization. Culture media [Neurobasal, B27, glutamine; Life Technologies, CA] containing growth factor (either IGF-1 or BDNF; Peprotech Inc., NJ) were added to the left channels of these devices (∼250 µL per device, 6 devices/condition), and media without growth factors was added to the right channel, to establish a gradient from the left channel to the right channel across the 3D gel within each device. Control devices received no growth factors. Devices were incubated at 37 °C in humidified CO_2_ incubators for 48 h. Cell cultures for receptor-staining studies were also performed under similar conditions (n = 3 per case).

### Immunostaining and quantification

Media was removed from the channels at the end of 48 h, cells fixed with 2% paraformaldehyde (10 min), washed with 1× PBS twice, incubated with 0.1% triton X-100 (Omnipur) for 5 min and rinsed with 1× PBS twice. Then 1% BSA (Sigma) solution was added to the wells and the devices incubated (37 °C, 30 min), followed by incubation with Alexa Fluor 488 or Alexa Fluor 594 Phalloidin (Life Technologies) for 20 min. Devices were washed three times with 1× PBS and imaged using an Axiovert A1 Zeiss epi-fluorescence microscope. Image analysis was done using NIH ImageJ software with the NeuronJ plugin, and neurite outgrowth and turning traced and quantified. Results within each test case were averaged across the devices and compared to controls under similar conditions. Neuronal viability under each case was also evaluated using LIVE/DEAD cell viability assay (Life Technologies). For receptor-staining studies, primary antibodies (IGF-IRα, TrkB) and serum were obtained from Santa Cruz Biotech, diluted 1∶200 in PBS, and appropriate secondary antibodies were purchased from Molecular Probes. Nuclear staining was visualized using DAPI (1∶10,000; Sigma). All statistical analysis was performed using SigmaPlot software via parametric and/or non-parametric analyses, as appropriate, with a minimum significance level set at *p*<0.05.

### Simulation methods

Although the neurons were cultured in 3D scaffolds within the microfluidic device in our experiments, COMSOL simulations showed little variation of chemogradient in *y*- and *z*- axes. Thus, the mathematical model we developed here is one-dimensional (1D). Furthermore, we assumed that the domain is only in the *x*-direction (*l* = 5 mm) and the growth cone migration depends only on the boundary concentration (*c_0_*>0) of the diffusing biomolecule, its diffusion coefficient, and the chemotaxis term (*χ*) in Eq. 3, within a 48 h time period. The partial differential equations (PDEs) were solved in MATLAB.

### Mathematical model

Let *c* be the chemical concentration and *g* be the growth cone density. We assume that the consumption of the chemical by the media and the cells is negligible. Then the PDE for *c* is
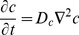
(1)where *D_c_* is the chemical diffusion coefficient. Based on the boundary and initial conditions for *c*, and taking *J_g_* as the flux of *g*, then
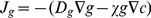
(2)where *D_g_* is the growth cone diffusion coefficient, and *χ* is the effect due to chemotaxis. The values of *χ* also takes into account the receptor-saturation phenomena typically dominant at higher *c*. It follows that
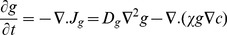
(3)


The described PDE system is a typical Keller-Segel model. For *g*, we assume no-flux conditions at the boundary points, and a Gaussian-shaped function centered at the center of the domain [44] with maximum value *g_0_* and matching the boundary conditions as the initial conditions. The dynamics that we would like to capture in our model is the same as that in the experiments, i.e., the movement of the center of mass of the growth cone. If we assume that there is just a single growth cone responding to the chemical gradient in the domain, then this center of mass will move a certain distance after time t. This experiment could be repeated several times to obtain the average distance moved by the center of mass of the growth cone due the effect of the chemical gradient. Thus, our model assumes that the initial distribution of the center of mass of the growth cone, which we refer to as the growth cone density, is Gaussian-shaped in the domain. This function is chosen so that the width at half-height is much smaller than the size of the domain of the PDE. The movement of the peak of this Gaussian-shaped curve due to the chemical gradient is comparable to the movement measured in the experiments performed, as shown in Fig. 1A (and Graphical Abstract S1). The negative sign for the chemotaxis term 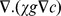 implies that movement of the growth cone is towards a higher chemical concentration.

Density of various cells as a continuous variable has been commonly treated in the literature for the past several years [44], [45]. Although discrete alternatives like agent based models could be used, they are typically unwieldy mathematically, and depend on interactions between the cells and the surrounding environment which cannot be easily measured or quantified. The diffusion equation is the limit of a stochastic process and a time-homogeneous Markov process; therefore, we believe it is reasonable to assume that the initial growth cone density is approximately Gaussian. We also assumed that any growth factor produced by the cells compensates for that degraded within the matrix, with no net effect.

In one dimension, the boundary concentration at *x* = 0 for *c* is held experimentally at a constant *c_0_*, and the flux of *c* at *x = l* is assumed to be zero, where *l* is the length of the gel region within the device. We assume that matrix concentration remains homogenous throughout the length *l*. The PDE for the chemical with the prescribed initial and boundary conditions has an exact solution:
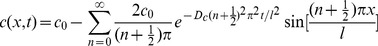
(4)


If *χ* is constant, the PDE for *g* is linear, and in this case, we may seek an exact solution for *g*. This would then imply that the distance (*d*) moved due to chemotaxis will continue to increase as the boundary concentration of the chemical (and thereby the gradient) increases. However, this is not supported experimentally, where increase in the boundary concentration *c_0_* has little chemotaxis effect beyond certain values, possibly due to receptor-saturation [46]. Following the form of *χ* used in [47], [48], and formally derived in [49], we take 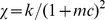, where *k* and *m* are parameters of the model. A typical graph of *χ(c)* is shown in Fig. 1B. It is clear that if *c*∼0, then *χ*∼*k*, so that *k* is the measure of chemotaxis for small *c*. Analogous to Michaelis-Menten kinetics [49], 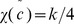, then 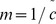. These parameters were derived from data fitting of our experimental findings. Since the model assumes that the parameters *k* and *m* are independent of the chemical concentration *c* and the density of the matrix, we determined *k* in all cases when *c_0_* = 0.1 µg/mL. Once *k* was obtained, we used data in the case when *c_0_* = 1 µg/mL and determined the parameter *m*. The form of the coefficient of chemotaxis in the model assumes that the overall effect of the chemical signal on the movement of the growth cone follows enzymatic kinetics [49]. This also confers the predictability of the model, but its elucidation via the signaling pathway is quite involved and beyond the scope of the current study.

For a time *t_1_*>*t_0_*, we define the distance moved due to chemotaxis in time *t_1_−t_0_* by *d =  |x_1_−x_0_|*, where *x_1_* is value of *x* where max *g(x, t_i_)* is attained, as illustrated in Fig. 1A. We emphasize that in all our simulations, *d*<<*l*. The values of parameter *k* is determined when *c_0_,* and hence *c(x, t)*, is small, and *m* is determined using larger values of *c_0_*. The values of *k* we estimated are comparable to those used in literature, within the same order of magnitude [45], [50]. Neuron turning is accounted for in the model by observing that *x_1_*<*x_0_*, and that *c(x_1_, t)* > *c(x_0_, t)* for all *t*, for the chemoattractants IGF-1 and BDNF.

## Results and Discussion

### Establishment of diffusive chemogradients within 3D scaffolds

**Figure 2:**
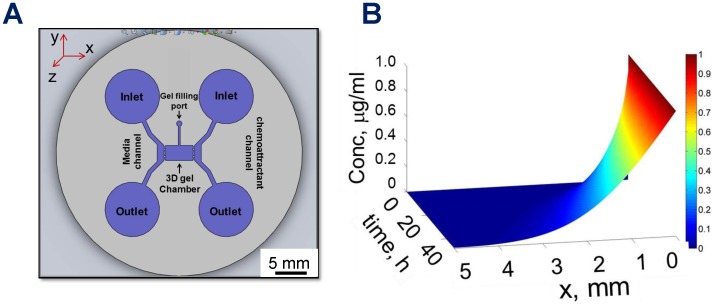
Microfluidic device design and gradient formation. (A) Schematic of the microfluidic device developed in this study, showing the gel chamber, inlet and outlet ports for media channels, and gel-filling port. (B) Spatio-temporal evolution of the concentration profiles in the gel region within the device over 48 h, for the conditions described above. Gradient was plotted within the gel region (5 mm), excluding the PDMS posts on both sides (300 µm×300 µm).

The microfluidic device (Fig. 2A) has separate gel-filling port, and two media channels to create chemogradient across the 3D scaffold of interest. The time-dependent snapshots of gradients across the gel within the device were obtained with a 20 kDa molecule (10 nM concentration) over a 48 h period, and the resulting concentration profiles showed that the gradient could be maintained for more than 4 days, as long as the solutions are replenished daily [40]. Theoretically, the time *τ* required for significant diffusion to establish over length *l* is given by *τ =  l^2^/(4π^2^D_c_)*, where *D_c_* is the diffusion coefficient of the chemical within 3D collagen gel [51]. For IGF-1, one of the cues used in our study, we used *D_c_* = 8×10^−12^ m^2^/s, which gives *τ*≈21 h, close to that observed experimentally (*Fig. S2*).

Studies have shown that the width of growth cones is ∼10 µm in 3D collagen gels [34]. Using experimental and computational approaches, Goodhill et al. suggested that growth cones could detect gradients as low as 0.1%, and exhibit robust guidance within 1-10% gradient steepness [36]. Defining steepness as the percentage change in concentration across 10 µm, our FEM modeling predicted an average steepness ranging between 0–5% during the 48 h time period at the concentrations studied, which should induce guidance as per earlier studies [52]. Growth cones experience a variety of gradients *in vivo*, and adapt their morphology and directional sense based on the magnitude of concentration and the gradient [53]. However, it is important to decouple the effects of growth factor concentration levels and gradients on cellular responses, as higher growth factor concentrations might saturate cell surface receptors resulting in deprivation of gradient effects, while lower concentrations might activate too few receptors despite higher gradients.

The spatio-temporal evolution of the concentration profiles within 3D scaffold over 48 h (20 kDa molecule) was shown in Fig. 2B, where the initial concentration in chemoattractant channel was set at 1 µM and that in cell and media channel to zero. Results from the gradient distribution (slope of the concentration profile) and its spatio-temporal evolution across the gel region (data not shown) were in agreement with experimental data on diffusion of FITC-dextran within this device. As discussed earlier, our experimental and computational data suggests diffusion and chemogradient to establish and progress predominantly along the *x*-axis, and so our mathematical framework had been developed in 1D. Similar concentration gradient analysis was performed for the 27 kDa molecule, and data analysis suggests that the 27 kDa molecule will diffuse 15% more slowly than the 17 kDa molecules, provided all other conditions remain constant. Our data also suggests that the chemogradients will primarily develop in the *x*-*y* plane without much variation in the *z-*axis at each slice within the 3D scaffold, since the scaffold height (150 µm) is significantly smaller than gel length (5 mm) or width (2.7 mm). To conclude, unlike conventional culture platforms (e.g., pipette-turning assay, gel transplant assay, Boyden chamber), the microfluidic platform developed here provides an exact knowledge of the concentration gradients within the 3D gel, and enables us to perform detailed parametric studies using simulations. This information is highly desirable for accurately assessing the precise chemogradients sensed by the growth cones to influence their decision making.

### Chemogradient affects neurite outgrowth and turning

**Figure 3:**
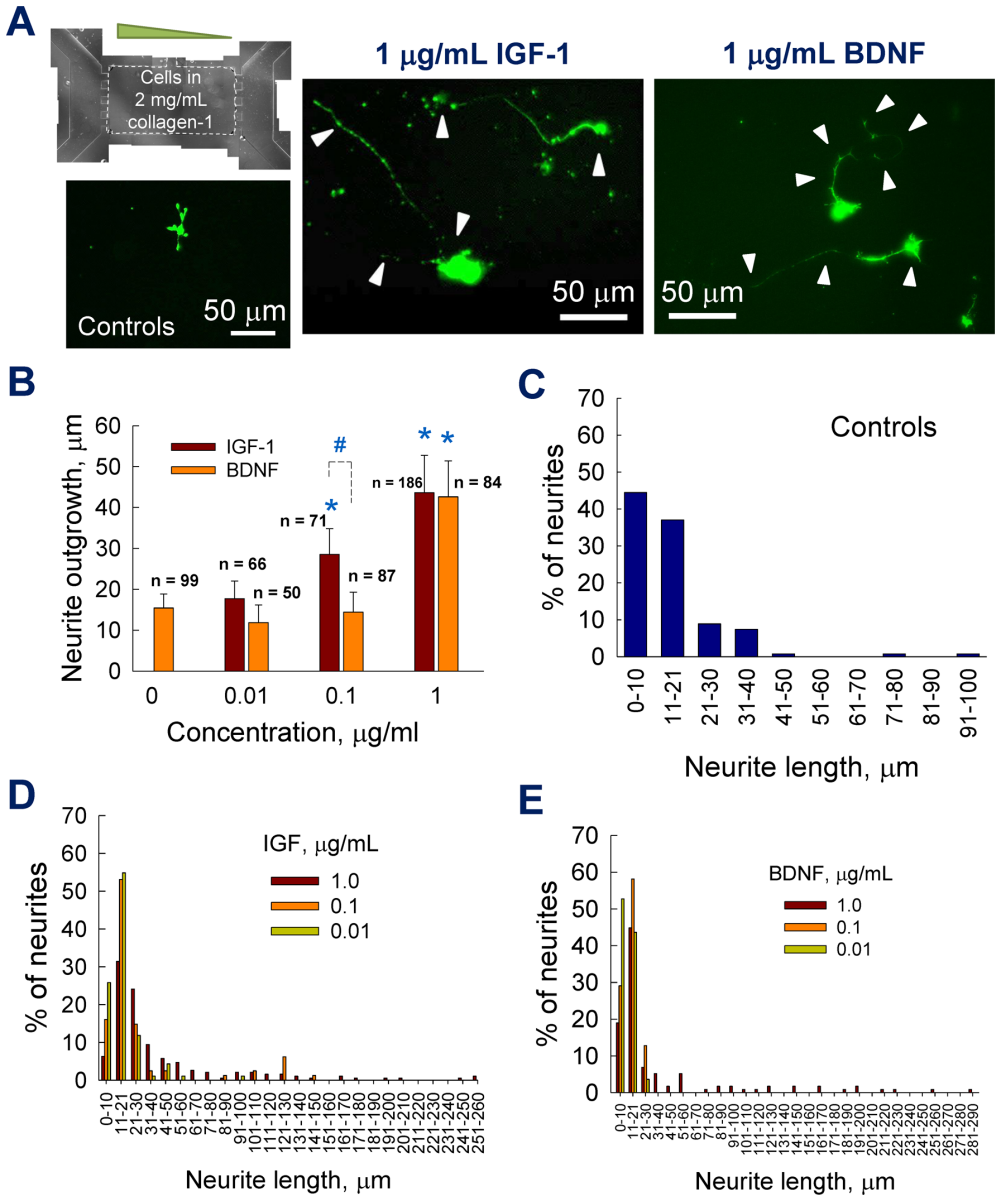
Quantification of neurite outgrowth within 2/mL collagen gels. (A) Montaged phase-contrast image of the gel region within the device. Cortical neurons are cultured within 3D collagen-1 gel (2 mg/mL) and chemogradient was established from left to right channels. Representative immunofluorescence images of the cortical neurons showing neurite extensions (pointed by arrowheads in selected cases) cultured under BDNF or IGF-1 gradients (0 and 1 µg/mL). The devices were fixed and stained at 48 h time point. (B) Average neurite outgrowths from images were quantified under respective conditions. * indicates significance in values (*p*<0.01) compared to controls, and ^#^ indicates significant differences (*p*<0.01) between those exposed to IGF-1 vs. BDNF at 0.1 µg/mL concentration. Neurite outgrowth distributions within controls (C), IGF-1 –treated cultures (D) and BDNF –treated cultures (E) were plotted to compare distinct differences in their outgrowth patterns. The percentage of neurites within each length bin was quantified and shown here, while their averages and standard deviations were shown in B.

Cortical neurite outgrowth within 2 mg/mL collagen scaffolds and in the presence of growth factors (IGF-1, BDNF) was highly influenced by the type and concentration of the chemogradient (Fig. 3A). Neurons were cultured for 48 h in collagen scaffolds, in the designated gel chamber within the microfluidic device (scaffold region identified by dotted lines), with chemogradient introduced in the left chamber and control medium in the right chamber. We stained for F-actin in this study to broadly identify the neurite outgrowth response to chemogradients. In the future studies, we will stain for tau protein and MAP2 separately, to identify and quantify individual responses of axons and dendrites respectively, to the same chemogradient. In contrast to the neurons obtained from induced differentiation of various stem cell types (neural, embryonic, iPSC, etc.), the cortical neurons studied here typically undergo relatively harsh procedures prior to their *in vitro* culture [40], [41], including isolation of embryonic brain tissue, enzymatic digestion to remove matrix, cell sorting, multiple centrifugation steps, etc. Therefore, it is desirable to evaluate the cellular responses to external factors such as chemogradients and scaffold characteristics within a limited time frame. Representative immunofluorescence images are shown in Fig. 3A, and the neurite outgrowth into three dimensions was evident in all the cases. A concentration-dependent neurite outgrowth was observed in the presence of either IGF-1 or BDNF, with higher concentrations eliciting more pronounced effect. The cell survival, as imaged and measured from cell viability assay (*Fig. S3*) appeared not affected by the concentration or type of growth factor studied. Average neurite outgrowth within control cultures, as quantified by the length of extending neurite from soma to the growth cone, was ∼15±3 µm (Fig. 3B). Although lower BDNF dosages (0.01 and 0.1 µg/mL) had no significant effect on the average neurite outgrowth (*p*>0.5 vs. controls), a robust 3-fold increase in neurite length was observed within 1 µg/mL-supplemented cultures (*p*<0.001 vs. controls; *p*<0.001 vs. lower dosages of BDNF). Similar to BDNF, IGF-1 at 0.01 µg/mL concentration had no additional effect on neurite outgrowth, relative to controls. At 0.1 and 1 µg/mL dosages, IGF-1 elicited 1.9-fold and 3-fold increases in average neurite outgrowth, respectively (*p*<0.001 vs. controls and 0.01 µg/mL IGF-1). The average neurite length over a 48 h culture period in this study is comparable with that reported in literature. For example, Kunze et al. reported average neurite outgrowth between 10–20 µm over a 2-day culture [54], and 70–90 µm over a 5-day culture [55].

We further analyzed the neurite outgrowth distributions within control and test cases. A majority of the neurites (∼85%) in control cultures had an outgrowth between 1–20 µm, with lower than 5% of neurites between 41–100 µm (Fig. 3C). A similar pattern was noticed within IGF-1 cultures at both 0.1 and 0.01 µg/mL, where 85% of the neurites were 1–30 µm in length (Fig. 3D). The maximum neurite outgrowth was 30 µm at both 0.01 and 0.1 µg/mL BDNF, and all the neurites were within 1–30 µm in length (Fig. 3E). On the other hand, neurite outgrowth distribution within 1 µg/mL IGF-1 or BDNF cultures was more spread, with at least 20% between 31–100 µm and another 10% of neurites within 101–290 µm range (statistical analysis for data in Figs. 3D and 3E described in the *Methods S1*).

**Figure 4:**
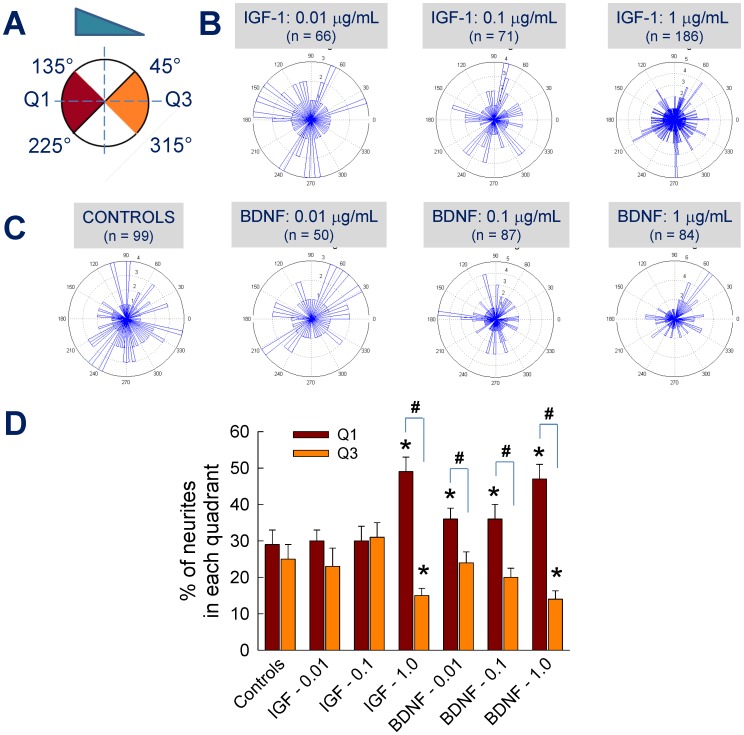
Quantification of neurite turning within 2/mL collagen gels. (A) Metrics for neurite turning quantification: neurons counted in red region (135°<Q1<225°) suggest turning towards gradient while those in orange region (45°>Q3>315°) represent turning away from gradient. Wind-rose plots of neurite turning under controls and BDNF (C), or IGF-1 (B) treated cases. Quantification of averaged neurite turning data under respective culture conditions (D) shows significant differences in neurite response within selected cases. * indicates significance in values (*p*<0.01) compared to controls, and ^#^ indicates significant differences (*p*<0.01) for Q1 vs. Q3 within each group.

In general, neurite orientation (Fig. 4A) between 135°–225° was deemed as turning towards the gradient (Q1), while that between 45°–315° was assessed as turning away from the gradient (Q3). Neurite orientation and turning in 2 mg/mL collagen scaffolds within control and test cases was quantified (Fig. 4D) from respective wind-rose plots (Fig. 4B, C). Within control cultures, as expected, the turning was more random, with ∼30% of neurites within Q1 and ∼26% within Q3. Compared to controls, addition of IGF-1 at 0.01 or 0.1 µg/mL did not influence neurite turning within 2 mg/mL collagen scaffolds (Fig. 4B). However, in the presence of 1 µg/mL IGF-1, 49% of the neurites were seen in Q1 while 14% turned towards Q3, suggesting the strong chemoattractive nature of IGF-1 at this dosage (*p*<0.001 vs. controls for both Q1 and Q3; *p*<0.001 for Q1 vs. Q3). On the other hand, even lower dosage of BDNF seems to have chemoattractive effects on cortical neurite orientation (Fig. 4C). Higher neurite turning towards Q1 was noticed at all tested doses of BDNF (*p*<0.01 vs. controls; *p*<0.01 vs. Q3), with maximum affect noticed at 1 µg/mL BDNF (*p*<0.01 vs. lower dosages). Although significant differences in neurite turning towards Q1 was noted between BDNF and IGF-1 at lower concentrations (*p*<0.01), 1 µg/mL concentrations elicited no such differences. Taken together, results show a strong dependence of neurite outgrowth, distribution and turning on concentration and type of biomolecular gradient within 2 mg/mL collagen-1 scaffolds.

Although adult neurons might possess distinct advantages (inexpensive, homogenous, etc.), embryonic primary neuron cultures are routinely performed in neurotoxicity and neuropharmacology studies because their extraction methods are relatively simple compared to adult neurons. IGF-1 and BDNF have been implicated to play important role in the nervous system and neurogenesis, under both healthy and inflammatory conditions, by regulating cell growth, differentiation, migration, survival and neurite outgrowth [56]–[58]. For example, IGF-1 was shown to regulate neurite outgrowth and cell migration from rat dorsal root ganglion explants in a dose-dependent manner (0–20 nM) [59]. Similarly, BDNF was found to significantly enhance cortical neurite outgrowth in a dose-dependent (0–100 pg/mL) manner when cultured in the presence of immature astrocytes, suggesting potential applications in axonal regrowth strategies after spinal cord injury [60]. The effect of diffusible and surface-bound BDNF gradient (50 ng/mL) on xenopus spinal neurons cultured on 2D laminin coated surfaces within microfluidic devices has been reported earlier [61]. However, the physiological concentration gradients and steepness of these molecules which regulate neurite outgrowth *in vivo* and *in vitro* remains unclear [62], [63]. Besides, these studies were performed on 2D substrates, which might not mimic *in vivo* cellular responses. Nevertheless, the intracellular signaling pathways by which BDNF or IGF-1 gradients regulate neurite outgrowth within cortical neuron 3D cultures remain elusive, and will be investigated in our future studies.

### Scaffold stiffness regulates neurite outgrowth and turning

**Figure 5:**
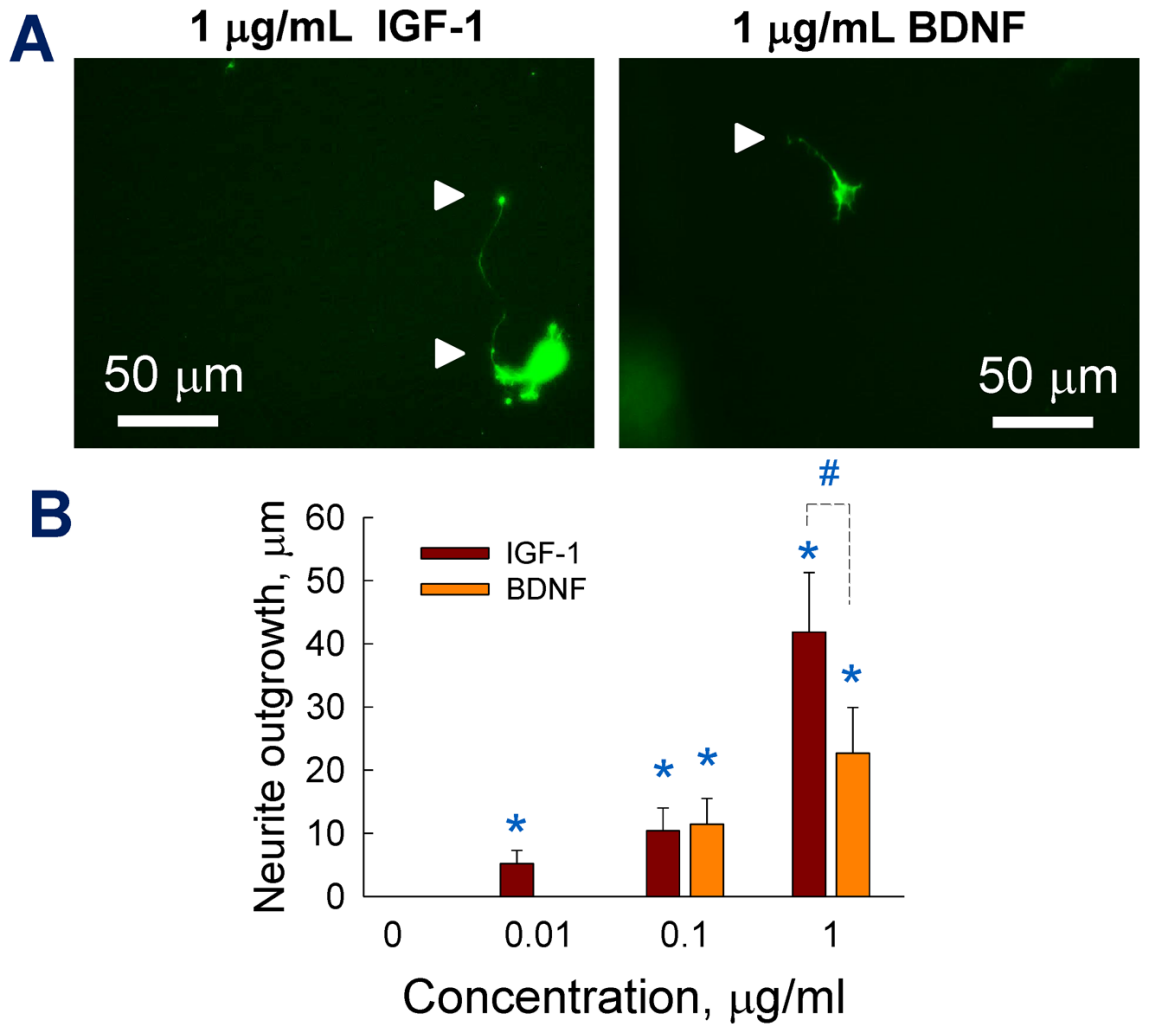
Quantification of neurite outgrowth within 1/mL collagen gels. (A) Representative immunofluorescence images of cortical neurons cultured within 1 mg/mL collagen-1 gels under IGF-1 or BDNF (1 µg/mL) gradients. (B) Neurite outgrowth data was quantified from such images. Meager average neurite outgrowth (<100 nm) was noticed within controls and 0.01 µg/mL BDNF cultures. * indicates significance in values (*p*<0.01) compared to controls, and ^#^ indicates significant difference (*p*<0.01) between cultures exposed to IGF-1 vs. BDNF at 1 µg/mL concentration. Significantly lower number of neurites could be counted in 1 mg/mL collagen-1 cultures compared to their 2 mg/mL counterparts.

One hypothesis for this study was that matrix stiffness mediates chemogradient-regulated neurite outgrowth and turning of cortical neurons. This was tested by culturing neurons within 1 mg/mL collagen scaffold within microfluidic devices, under conditions similar to that for 2 mg/mL collagen, and quantifying neurite outgrowth. Representative immunofluorescence images of cultures in selective cases are shown in Fig. 5A. Neurite outgrowth was barely noticeable within controls and at low concentrations of IGF-1 and BDNF. At 1 µg/mL concentration, significant neurite outgrowth into 3D scaffold was noticed. Quantifiable neurite outgrowth was not evident within controls and 0.01 µg/mL BDNF cultures (Fig. 5B), although 0.01 µg/mL IGF-1 stimulated ∼5 µm average neurite outgrowth (*p*<0.01 vs. controls; *p*<0.001 vs. 0.01 µg/mL BDNF). Neurite outgrowth in cultures receiving 0.1 µg/mL IGF-1 or BDNF was similar, and significantly higher than that in control (*p*<0.001) or within 0.01 µg/mL counterparts (*p*<0.001). At higher concentration (1 µg/mL), IGF-1 stimulated an average neurite outgrowth of 42±9 µm, which is 4-fold higher than that within 0.1 µg/mL IGF-1 cultures (*p*<0.001), 8-fold higher than that within 0.01 µg/mL IGF-1 cultures (*p*<0.001), and 2-fold higher than that within BDNF cultures at 1 µg/mL concentration (22±7 µm; *p*<0.01). In general, cortical neurite outgrowth within 1 mg/mL collagen scaffolds, in the presence of 0–0.1 µg/mL BDNF or IGF-1, was significantly lower than their counterparts within 2 mg/mL collagen scaffolds (*p*<0.001 in all the cases). Interestingly, neurite outgrowth within 1 µg/mL IGF-1 cultures appears independent of the collagen concentration (and thereby stiffness, porosity, pore-size, etc.), while lowering collagen concentration inhibited neurite outgrowth within 1 µg/mL BDNF cultures.

The reconstituted type I collagen used in this study exhibits varying physical properties and mechanical moduli, depending on the pH or concentration at which it is prepared [64]. Although collagen is not the predominant ECM component in the CNS, type I collagen has been routinely used by researchers to study neurite outgrowth in both 2D and 3D cultures [65]. This is because, type I collagen might be relatively inert, and may not interfere with axonal activity or binding of diffusing signaling molecule, etc. Secondly, from a regenerative medicine or tissue engineering stand point, it is relatively simple and economical to make collagen gels, consistently with high fidelity, to test feasibility of such bioengineering approaches. Therefore, in this study, we also tested the utility of collagen gels, to be able to compare our outcomes with that in literature. Yang et al. have shown that at 37 °C, by increasing collagen gel concentration from 1 mg/mL to 2 mg/mL, pore size was halved, fibril size was doubled, and gel storage modulus (*G′*) increased (scaled as *c^2.1^*, *c* being gel concentration) [66]. We propose that lower (or complete absence) neurite outgrowth within 1 mg/mL collagen scaffolds could be due to the significantly compromised gel rigidity, coupled with higher pore size and thinner fibril size, contributing to lower number of reliable adhesion sites available for the growth cones to attach, interact and navigate. Although steady-state gradients within 1 mg/mL and 2 mg/mL collagen scaffolds would be similar (>40 days), our data suggests that it will take longer time for the gradients to be established within 2 mg/mL collagen scaffolds (>40 h). To further validate our underlying hypothesis, current experiments are aimed at quantifying cortical neurite response to chemogradients within collagen scaffolds of different concentration (1.5 mg/mL, 2.5 mg/mL, 3 mg/mL, etc.). In all cases, we were easily able to obtain parameter estimates in the mathematical model using the experimental data we obtained.

### Scaffold composition mediates neurite responses

**Figure 6:**
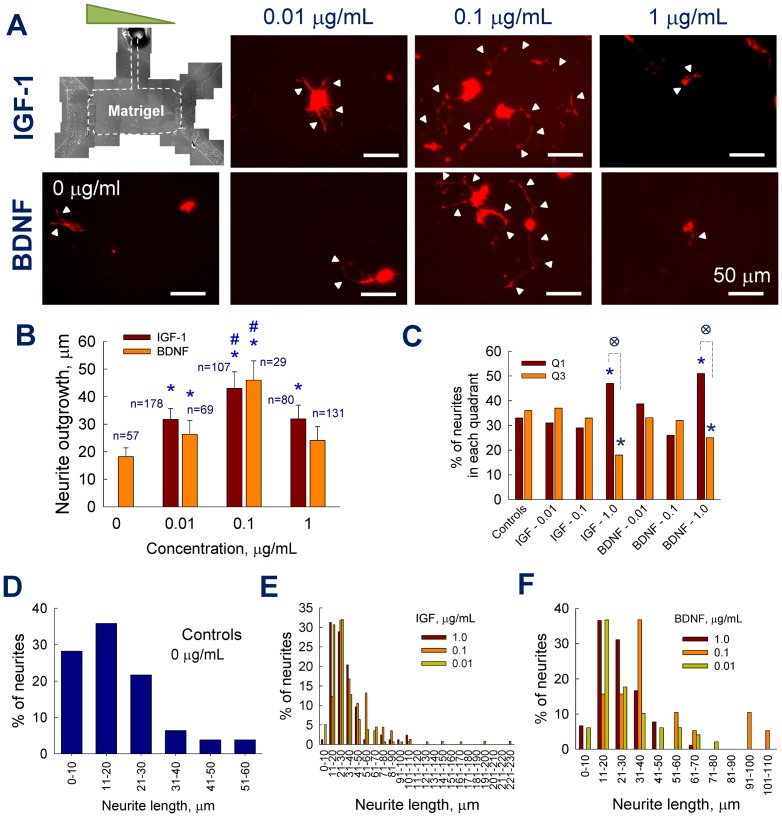
Quantification of neurite outgrowth and turning within matrigel. (A) Montaged phase-contrast image of the matrigel-filled region within the device. Cortical neurons were cultured within 3D matrigel (as-received) and chemogradient was established from left to right channels. Representative immunofluorescence images of the cortical neurons showing neurite extensions (pointed by arrowheads in selected cases) under BDNF or IGF-1 gradients (0–1 µg/mL). The devices were fixed and stained at 48 h time point. Average neurite outgrowth (B) and turning (C) were quantified from images taken under respective conditions. Neurite outgrowth distributions within controls (D), IGF-1 –treated cultures (E) and BDNF –treated cultures (F) are shown for comparison. * indicates significance in values (*p*<0.01) compared to controls; ^#^ indicates significant differences (*p*<0.01) between 0.1 µg/mL and other concentrations within IGF-1 or BDNF cultures; **^∶^** indicates significant differences (*p*<0.01) for Q1 vs. Q3 within 1 µg/mL IGF-1 and BDNF groups.

Within microfluidic devices, neurite outgrowth and turning in as-received 3D matrigel (contains laminin, collagen-IV, entactin and heparin sulfate) scaffolds was evaluated in the presence or absence of chemogradients (Fig. 6A). Immunofluorescence images reveal that compared to controls, neurite extensions significantly increased within 0.01 and 0.1 µg/mL cultures under both IGF-1 and BDNF gradients. Although IGF-1 or BDNF at 1 µg/mL dosage stimulated higher neurite outgrowth compared to controls, they appeared to suppress neurite outgrowth compared to that at 0.1 µg/mL dosage. This data is in contrast with our observations within collagen scaffolds, where significant neurite outgrowth was evident at higher concentrations of IGF-1 gradients. While the average neurite outgrowth was 18±4 µm within control cultures, it increased by 1.6±0.3 –fold, 2.4±0.4 –fold and 1.6±0.5 –fold within 0.01, 0.1 and 1 µg/mL IGF-1 cultures, respectively (Fig. 6B; *p*<0.01 vs. controls in all the cases). Although 1 µg/mL BDNF offered no additional advantage to neurite outgrowth compared to its absence (*p*>0.4), both 0.01 and 0.1 µg/mL BDNF stimulated significant neurite outgrowth (*p*<0.001 vs. controls; *p*<0.01 for 0.01 vs. 0.1 µg/mL BDNF), similar to that observed in their IGF-1 counterparts. Interestingly, no significant differences were observed in the average neurite outgrowth within control cultures, in matrigel and 2 mg/mL collagen-1 scaffolds (*p*>0.7). Conversely, neurite outgrowth within 0.01 and 0.1 µg/mL cultures (either IGF-1 or BDNF) was significantly higher within matrigel scaffolds, compared to 1 mg/mL or 2 mg/mL collagen-1 scaffolds (*p*<0.001 for matrigel vs. collagen). On the other hand, in the presence of 1 µg/mL IGF-1 or BDNF, neurite outgrowth within 2 mg/mL collagen was significantly higher than their counterparts in 1 mg/mL collagen or matrigel scaffolds (*p*<0.001). Within 1 µg/mL IGF-additive cultures, collagen scaffolds seem to support higher neurite outgrowth compared to matrigel (*p*<0.01 for collagen vs. matrigel), while the benefit of 1 µg/mL BDNF is higher in the presence of 2 mg/mL collagen alone.

Neurite outgrowth distribution analysis showed that ∼85% neurites in control cultures were within 1–30 µm length, with the rest within 31–60 µm range (Fig. 6D). Similarly, almost 80% neurites in 1 µg/mL and 0.01 µg/mL IGF-additive cultures were within 1–40 µm range (Fig. 6E). On the other hand, neurite outgrowth within 0.1 µg/mL IGF-1 cultures was more spread, with at least 50% of neurites within 31–90 µm range. Similar trends in neurite outgrowth distributions were observed in BDNF-additive cultures within matrigel scaffolds (Fig. 6F). As expected, neurite turning and orientation within control cultures was random, with equal number of neurites turning towards Q1 or Q3 (Fig. 6C; 33% and 36% respectively). Similar trends were noticed within both 0.01 and 0.1 µg/mL IGF-1 and BDNF cultures. Compared to neurite turning towards quadrant Q3, significantly higher turning towards Q1 was noticed within 1 µg/mL IGF and 1 µg/mL BDNF cultures (*p*<0.01 for Q1 vs. Q3 in both the cases; *p*<0.01 vs. controls for Q1 and Q3 in both the cases). Interestingly, although 0.1 µg/mL IGF-1 and BDNF promoted relatively higher neurite outgrowth compared to other cases, they could not influence significant turning towards the gradient (Q1). On the other hand, both IGF-1 and BDNF at 1 µg/mL dosage stimulated significant number of neurites to turn towards the gradient (*p*<0.001 for Q1 vs. Q3). We hypothesize that 1 µg/mL concentration steered sprouting neurites towards the gradient during the initial stages of neuritogenesis, after which receptor-saturation might have curtailed growth cone migration (and thereby neurite extensions) within these cultures.

Compared to our earlier report [40], lower neurite outgrowth observed in this study could be attributed to the following differences: (a) Cortical neurons used in this study could result in significantly different neurite responses, compared to hippocampal neurons and dorsal root ganglion neurons used in our previous study; (b) While 2 mg/mL collagen alone was utilized earlier, here we also tested the role of 1 mg/mL collagen and as-received matrigel. The differences in scaffold stiffness and composition could have contributed to various neurite responses; (c) In the previous study, cells were packed outside the 3D scaffold and interfaced to hydrogel using mild hydrostatic pressure, so that axons invade the 3D matrix in response to chemogradient. Here, we cultured cells within 3D scaffold, and so cells are in a different mechano-sensitive environment; (d) Finally, hippocampal neurons were exposed to different signaling molecules in our previous study (Netrin-1, Slit-2, etc.), while they were exposed to IGF-1 and BDNF here, which might be eliciting different responses from cortical neurons.

Numerous studies have shown that matrigel (as received, 10–12 mg/mL) forms a slightly dense gel, with pore characteristics slightly lower than that of collagen-1 scaffolds prepared at much lower concentration (2 mg/mL). While the pore size and porosity of matrigel might not facilitate cell migration, they do allow axonal and dendrite invasion through the open spaces. However, it is not clear at this stage if the growth cones generate any gelatinases or enzymes to break down matrix. We recently showed that the average modulus of as-received matrigel is 896±265 Pa, while that of 2 mg/mL collagen-1 (pH∼7.4) is 511±142 Pa [67]. As we reported earlier [67], the diverse protein composition of these matrices might be stimulating cortical neurons and their growth cones to express different sets of integrin subunits, which could have contributed to the observed differences in neurite outgrowth and turning within these two scaffolds.

### Model parameters estimation and their effects

**Figure 7:**
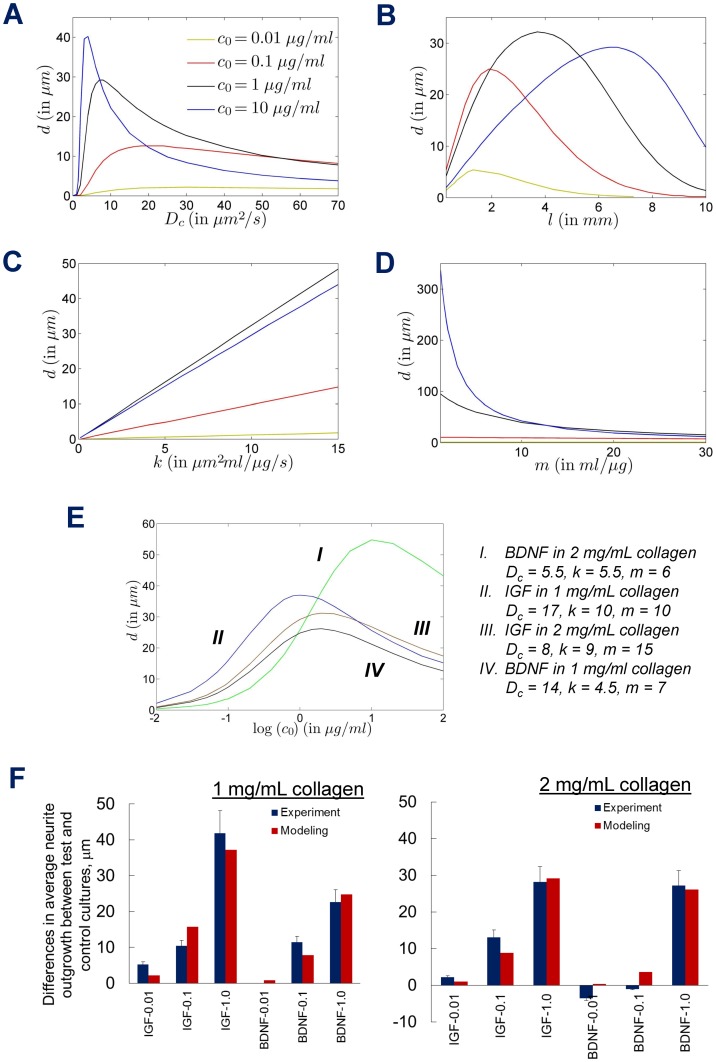
Mathematical modeling of neurite outgrowth. The effect of various parameters of the model on the distance traveled by growth cones (neurite outgrowth), when the boundary concentration *c_0_* is set at 0.01, 0.1, 1 and 10 µg/mL. The curves at *c_0_* = 10 µg/mL demonstrate the predictability power of this model. (A–D) Effects of the diffusion coefficient (*D_c_*), length (*l*) of the domain, and the chemotaxis parameters *k* and *m*, on growth cone movement. Unless otherwise mentioned, the parameter values are *D_c_* = 8 µm^2^/s, *l* = 5 mm, *k* = 9 µm^2^mL/µg/s, and *m* = 15 mL/µg. (E) Effect of the boundary concentration *c_0_* on the distance moved by the growth cone for different sets of culture conditions (BDNF or IGF, 1 mg/mL or 2 mg/mL collagen) investigated in this study. The respective parameter values of *D_c_*, *k* and *m* are provided for each curve. (F) Strong correlation between simulation results and experimentally-observed data was noted for the average neurite outgrowth under respective culture conditions.

We developed a mathematical model of chemogradient-mediated neurite outgrowth as a tool to generate and test hypotheses regarding the mechanisms behind biomolecular effects on cortical neuron behavior in 3D scaffolds. Our model significantly differs from previous mathematical models (e.g., modeling growth cone motility based on Brownian motion) on the role of growth cone dynamics in neurite outgrowth and guidance [68]. In the current model, the diffusion coefficients were estimated using the physical properties of the scaffold and the diffusing chemical, and the chemotaxis parameters were estimated using experimental data. The model simulations suggest that the effects of chemogradients on growth cone migration could be in large part mediated by *c_0_* and *D_c_* of the diffusing molecule. The effect of the various parameters of the model was shown in Fig. 7. The diffusion coefficient (*D_c_*) in each case was calculated using Stokes-Einstein equation, taking into account the molecular weight of the diffusing molecule and viscosity of the matrix through which the molecule is diffusing. Although Stokes-Einstein is more suitable to calculate *D_c_* of a molecule within simple fluids, it has recently been successfully used to estimate *D_c_* of molecules diffusing through viscous polymeric solutions [69], [70]. Furthermore, *D_c_* varies within CNS microenvironment as a function of local matrix properties (e.g., temperature, stiffness, density, porosity, pore size, fibril length, composition, cell density), and therefore critically regulates the effects of *c_0_*. While it would be ideal to incorporate each of these effects separately in the model, it is challenging to get experimental data for each of these individual effects, and we are unaware of such data in the literature. The model was applied to two cases: role of diffusing IGF-1 or BDNF within 1 mg/mL collagen or 2 mg/mL collagen scaffolds. It should be noted that the neurite outgrowth *(d)* computed from simulations (Fig. 7) corresponds to the differences in average neurite outgrowth between test and control cultures. As shown below, by adjusting the model parameters, we were able to fit the model simulations to experimental data.

Unless otherwise indicated, the parameter values for the plots are *D_c_* = 8 µm^2^/s, *l* = 5 mm, *k* = 9 µm^2^.mL/µg/s, *m* = 15 mL/µg and *D_g_* = 7×10^−3^ µm^2^/s [44]. Even without doing a formal sensitivity analysis, it can be seen from Fig. 7A that (i) for a given *c_0_*, neurite outgrowth is higher in scaffolds with higher *D_c_*, till it reaches a maximum for that particular *c_0_*, beyond which the *D_c_* effect subsides; (ii) with increasing *c_0_*, neurite outgrowth peaks within scaffolds at lower *D_c_* values; and (iii) the effect on the growth is the most sensitive if *c_0_* is 10 µg/mL, and 0<*D_c_*<10 µm^2^/s (Fig. 7A). It can be further deduced that maximum neurite outgrowth could also be achieved (i) by fine-tuning the properties (porosity, pore-size) of the matrix (*D_c_*) through which the molecule diffuses at a given *c_0_*, or (ii) by increasing *c_0_* within a scaffold whose *D_c_* is held constant. The only deviation we observed was for *c_0_* = 10 µg/mL and *D_c_*>10 µm^2^/s, and so an accurate estimate of the diffusion coefficient, especially in dense media and at high chemical concentration, is required in order to estimate the precise effect of chemotaxis in the model.

If all other parameters are identified *a priori*, and the boundary concentration *c_0_* is fixed, then we can identify the optimum gel region length (*l)* which facilitates maximum average neurite outgrowth (Fig. 7B). It is apparent that the average neurite outgrowth at a particular *c_0_* is higher within devices containing smaller gel region. This data clearly predicts the domain range over which a guidance cue could impart significant effect on neurite outgrowth and turning, which has potential applications in the design of neural tissue engineering scaffolds or drug delivery platforms. Although the effect of various factors (concentration, gradient steepness, inherent systemic noise, growth cone diameter, number of receptors, etc.) that affect neurite outgrowth have been modeled with varying success [15], [36], [53], [71], the specific effects of *D_c_* and *l* on neurite outgrowth had not been reported in such detail.

The effect of varying the chemotaxis parameter *k*, while keeping the other variables fixed was also assessed (Fig. 7C). In this case, at a given *c_0_*, varying *k* had a linear effect on the neurite outgrowth, because the model is almost linear for the choice of parameters. However, for the given set of parameters, we observed that increasing *c_0_* beyond 1 µg/mL offered no additional benefit to neurite outgrowth, for all values of *k*. On the hand, the effect of chemotaxis parameter *m* on the average distance moved by growth cones is more pronounced at lower values and at larger chemical concentrations (*c_0_*≥1 µg/mL; Fig. 7D). A dramatic increase in neurite outgrowth was noticed as *c_0_* was increased (0.01–10 µg/mL) at lower *m* values (<5 mL/µg), although such effects are not observed at higher *m*. Besides, if we know experimentally that the distance moved by the growth cone is not large, we can safely conclude that *m* is bounded away from zero for large values of the boundary chemical concentration *c_0_*. The dissociation constant (*K_d_*) for IGF-1 in human colon cancer cells was reported as ∼1–2 nM [72], while that for BDNF on embryonic chick neurons as ∼1 nM [73]. These values closely match the effective range of *m* (taking *m* ∝ 1/*K_d_*) computed for IGF-1 and BDNF in this study. Varying *D_g_* in the physiological range does not have any significant effect on the distance moved by the neurites (data not shown). We found that in the case of collagen, the chemotaxis parameters used in the model were only dependent on the growth factor, and was more or less independent of the characteristics of the scaffold.

We then evaluated the effect of varying *c_0_* (over four orders of magnitude) on neurite outgrowth within the experimental conditions in this study (Fig. 7E). For each case, the values of *D_c_*, *k* and *m* are shown for comparison. We first simulated the neurite outgrowth for IGF-1 and BDNF diffusing through 1 mg/mL collagen scaffolds. Based on the *k* and *m* values obtained for IGF-1 and BDNF, we then computed the neurite outgrowth within 2 mg/mL collagen scaffolds over a wide range of *c_0_*, by just varying *D_c_* values (Fig. 7E). Except for BDNF diffusing through 2 mg/mL collagen, it can be seen that the chemotaxis effect does not vary significantly for *c_0_*>10 µg/mL (possibly due to receptor saturation), for the sets of obtained parameter values. Crucially, we see that in all cases, our model predicts that an increase in *c_0_* does not produce an indefinite increase in the distance moved by the growth cones. Even without the typically associated stochastic effects, we see that the curves can still be distinguished because they correspond to significantly different *D_c_* values. Similarly, we estimated that *D_c_* = 4 µm^2^/s, *k* = 350 µm^2^.mL/µg/s, and *m* = 1100 mL/µg, for IGF-1 diffusing through matrigel scaffold. This indicates that the receptor activity within matrigel might be significantly different from that within collagen scaffolds, possibly due to additional signaling from matrigel protein components. Fig. 7F shows a comparison of the data from experimental results and model simulations, under respective conditions. The predicted average neurite outgrowths (*d*) at various *c_0_* (0.01–1 µg/mL) from these parameter values were in excellent agreement with that observed experimentally (Fig. 6B). We had earlier [74] shown that the average neurite outgrowth within 2 mg/mL collagen at *c_0_* = 10 µg/mL IGF-1 is ∼30 µm, which is very close to that predicted from our model (Fig. 7A–D). The predictability of the model is strongly reflected in the fact that by changing only the diffusion coefficient *D_c_*, and hence accounting for the change in the density of the matrix, and keeping the chemotaxis parameters more or less fixed, the model is able to account for the differences in the neuronal outgrowth (Figs. 7E, F). In conclusion, the average neurite outgrowth (under various conditions) predicted from our model closely matches the experimental values from microfluidic experiments, validating the salient prognostic feature of our model.

Although Keller-Segel model had been successfully used to describe chemotaxis of bacteria such as *E. coli*, eukaryotic cells (or growth cones in this study) move by deformation (extension and retraction of filopodia or lamellopodia) under chemical gradients, which makes the theoretical description and an understanding of the biological pathways involved, more complicated [75], [76]. Due to their relatively small size, bacteria were found to respond and migrate in a controlled fashion due to temporal differences alone in chemogradients, which could partially be explained using stochastic models [77]. Eukaryotic cells and growth cones, on the other hand, respond to both spatial and temporal variations in gradient, primarily by adapting to the 3D gel stiffness and composition [78]. Thus, in this study, we adapted Keller-Segel model to deterministically quantify neurite outgrowth by also incorporating the effects of *D_c_* within a 3D scaffold, and the domain length *l* over which the molecule is diffusing. We are currently extending this mathematical framework to quantitatively predict neurite turning under chemogradients within 3D scaffolds. Although further modeling and experimental data are needed to expand our model to include the effects of other parameters (e.g., time-dependent outgrowth, multiple simultaneous gradients), this study identifies the critical importance of *c_0_*, *l* and *D_c_* in estimating and predicting neurite outgrowth. Outcomes from this study could be of tremendous use in optimizing (i) dimensions, topography and composition of tissue engineered constructs, (ii) drug dosage for regenerative medicine purposes, and (iii) therapeutic cell transplantation approaches to treat various neurodegenerative injuries or disorders.

### Activation of cell surface receptors

**Figure 8:**
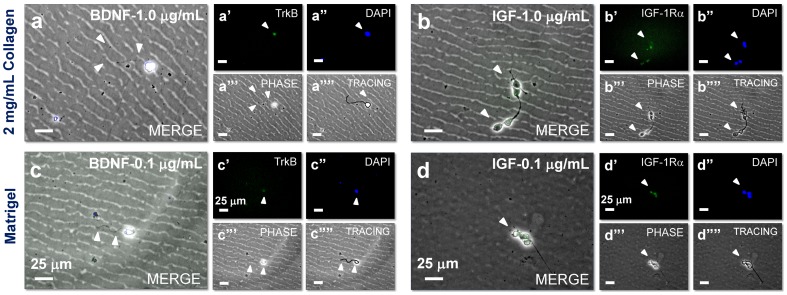
Effect of various culture conditions on receptor expression. Receptor expressions (TrkB and IGF-1Rα) on cortical neurons (selected cases for highest neurite outgrowth) in response to respective chemogradients (BDNF and IGF-1) within the microfluidic device. (a-d) Merged images showing receptor activation on cell surface (pointed by arrowheads) when cultured within 2 mg/mL collagen-1 or matrigel scaffolds, in the presence of BDNF or IGF-1 gradients, at 0.1 or 1 µg/mL initial concentrations. Individual receptor expression images (a*′*–d*′*), cell nuclei staining (a”–d”) and phase-contrast (a”’–d”’) under respective conditions are shown. Cell tracings within 3D scaffolds under respective conditions (a””–d””) have been included to aid visualization. Scale bars: 25 µm.

These experimental and modeling results led us to further explore the underlying molecular basis for cortical neuron response to chemogradients within 3D scaffolds. Neurotrophins mediate diverse functions in the CNS by binding to two kinds of receptors: tropomyosin receptor kinase (Trk) family and p75NTR. Dontchev et al. noted that BDNF addition stimulated higher growth cone activity and axonal extension in mice cortical neurons, possibly via involvement of NMDA receptors and Ca^2+^ flux-mediated receptor interactions [79]. BDNF regulates cell migration within developing cerebral cortex via TrkB signaling and P-13 kinase activation [80]. Activation and expression of IGF-1Rα was shown to be a requisite for axonal regeneration within adult CNS post-trauma [81]. We therefore tested whether the observed cortical neurite outgrowth and turning in response to chemogradients within 3D scaffolds is facilitated by respective cell-surface receptors (Fig. 8). Based on the neurite response data under 1 µg/mL BNDF or IGF-1 gradients within 2 mg/mL collagen scaffold, and under 0.1 µg/mL BDNF or IGF-1 gradients within matrigel scaffold, we stained for TrkB and IGF-1Rα receptors on cell surface within respective cultures. For each of the conditions tested, representative merged phase-contrast images were shown (Fig. 8), supported by the staining for location of nuclei (DAPI), staining for respective receptor on cell surface, and tracing of the cell body and axonal outgrowth in 3D (planes out of focus). Data for control cultures (with no growth factor gradient) was not shown here, as no receptor staining was observed. It could be seen from the images in Fig. 8 that significant receptor expression on cell surface was evident in response to growth factor gradients, which could be stimulating the cells to extend neurite outgrowth and turning under respective conditions. Further molecular biology studies (e.g., western blots, PCR analysis) to assess changes in such receptor expression at both transcriptional and translational levels might help support these results.

## Conclusions

Our study highlights how interactions between experiments and mathematical modeling could be complimentary for generating and testing hypotheses. Although the present paper is focused on neurite outgrowth and turning, this process is only one facet of neural circuit formation, which involves a wide range of processes including sequential differentiation of resident stem cells, cell migration, and cell-cell and cell-matrix interactions. The microfluidic platform demonstrated here could be used to study some of these phenomena *in vitro*, either in isolation or in combination with other variables. Future work on these aspects of neuritogenesis could provide an opportunity to further generalize this model for predicting and testing the effect of potential therapeutic targets. Such a robust model-driven parametric estimation approach could have potential applications in the mechanistic understanding of angiogenesis, cancer cell migration, stem cell differentiation and in neural tissue engineering.

## Supporting Information

Figure S1 **Dimensions of the microfluidic device.** Schematic of the microfluidic device used in this study, depicting dimensions of the media channels, ports, and gel chamber.
(TIF)

Figure S2 **Analysis of diffusion profiles in 2 mg/mL collagen gel.** (A) Time-lapse images of florescent FITC-dextran (10 nM, 20 kDa) diffusion through 2 mg/mL collagen gel within the microfluidic device. Quantification of the gradient along the gel revealed diffusion profiles similar to that from COMSOL simulations (B).
(TIF)

Figure S3 **Cortical neuron survival within collagen gels under molecular gradients.** Representative images of cell viability data from LIVE/DEAD assay, demonstrating high cortical neuron survival when cultured within 3D collagen scaffolds, and exposed to gradients of growth factor gradients for 48 h. Scale bar: 50 µm.
(TIF)

Methods S1 **Supporting data analysis.** Statistical analysis of the data shown in Fig. 3D (Table A) and Fig. 3E (Table B). The model parameters and their respective values under various conditions tested in this study are also shown (Table C).
(DOCX)

Graphical Abstract S1 (TIF)
