# Supplementary figures and images for: Synergistic Effects of 3D ECM and Chemogradients on Neurite Outgrowth and Guidance: A Simple Modeling and Microfluidic Framework
Source: PLoS One. 2014 Jun 10;9(6):e99640. doi: 10.1371/journal.pone.0099640 (PMC4051856; doi:10.1371/journal.pone.0099640)

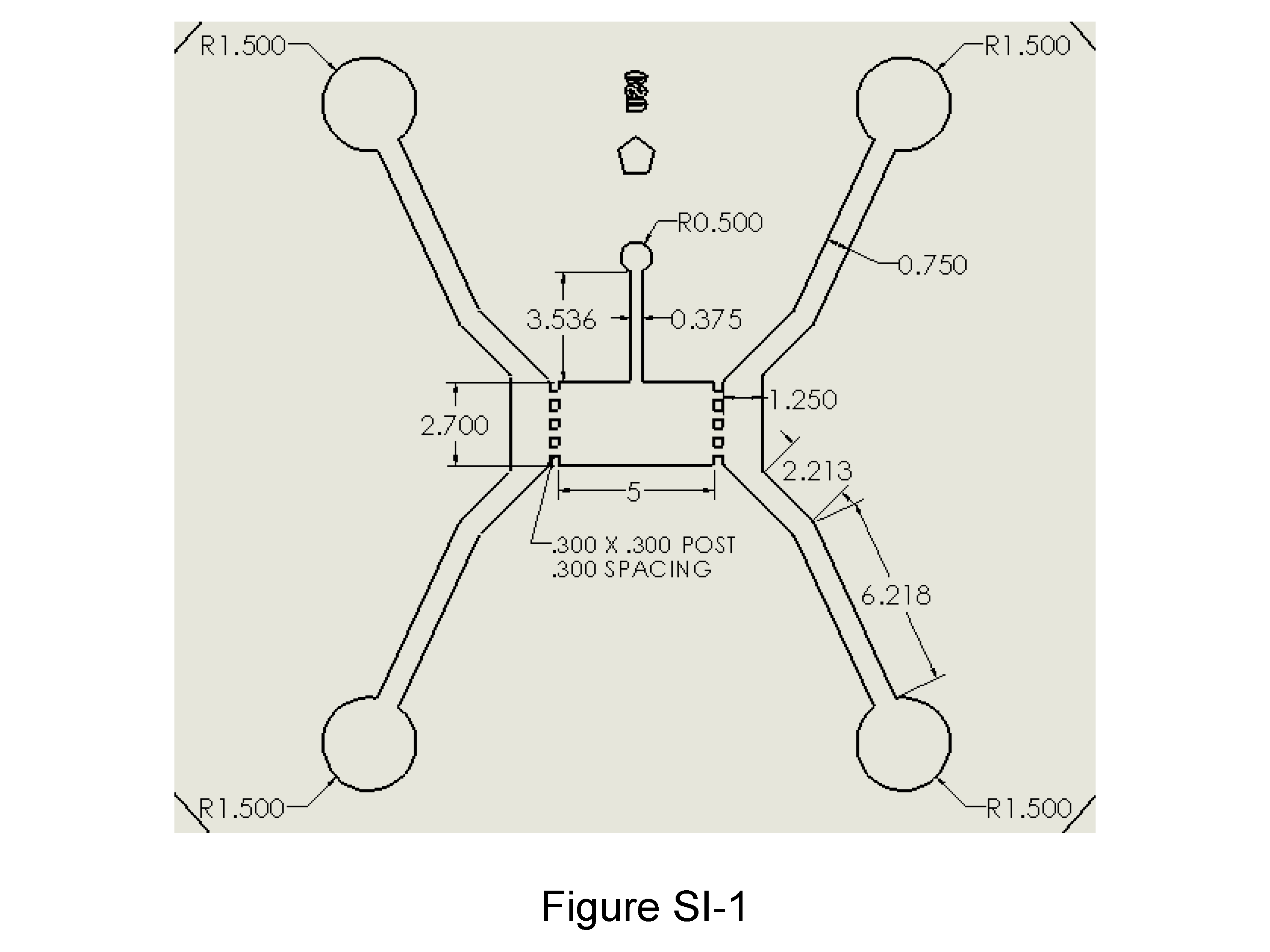

Supplement: Figure S1 — Dimensions of the microfluidic device. Schematic of the microfluidic device used in this study, depicting dimensions of the media channels, ports, and gel chamber. (TIF) [file pone.0099640.s001.tif]

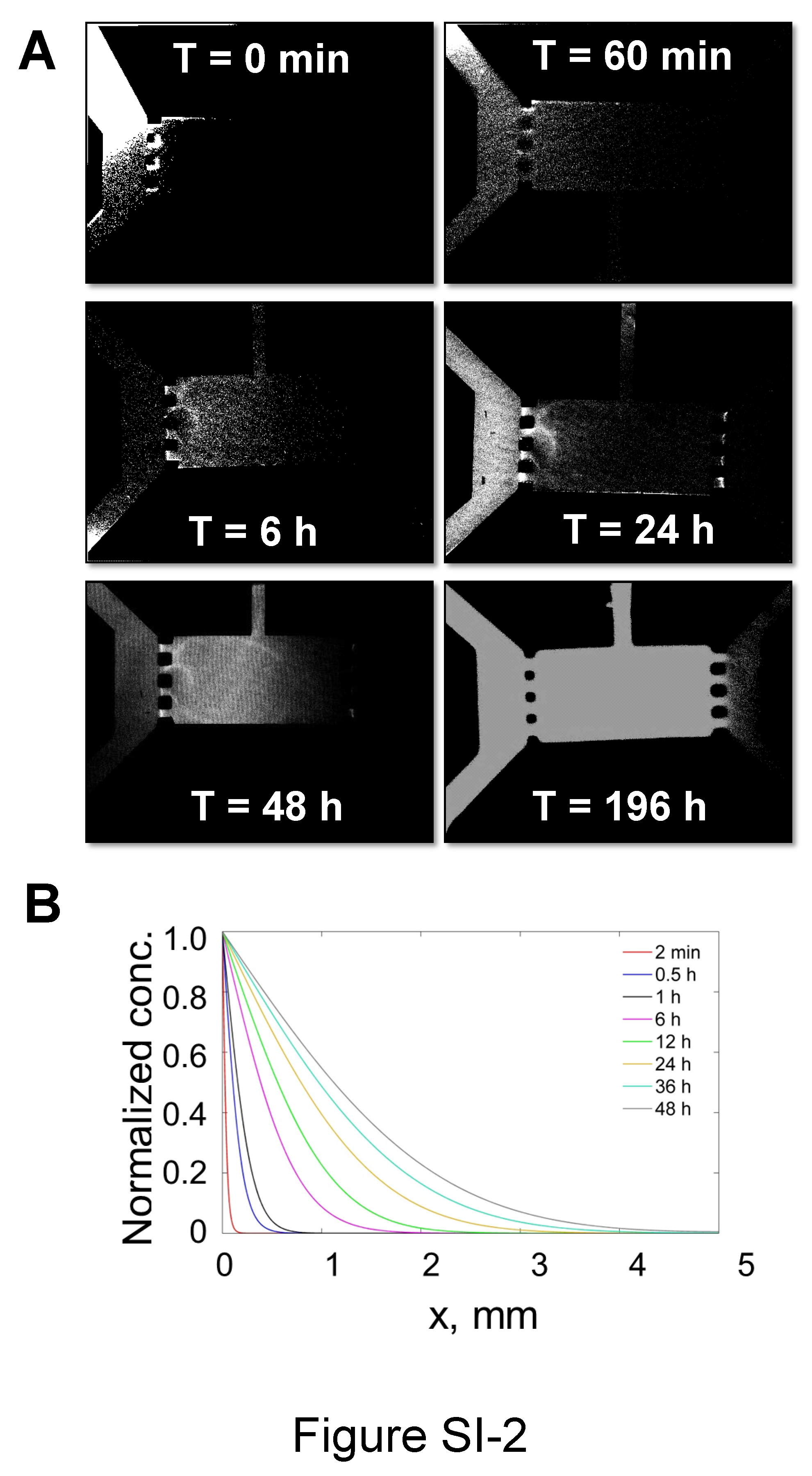

Supplement: Figure S2 — Analysis of diffusion profiles in 2 mg/mL collagen gel. (A) Time-lapse images of florescent FITC-dextran (10 nM, 20 kDa) diffusion through 2 mg/mL collagen gel within the microfluidic device. Quantification of the gradient along the gel revealed diffusion profiles similar to that from COMSOL simulations (B). (TIF) [file pone.0099640.s002.tif]

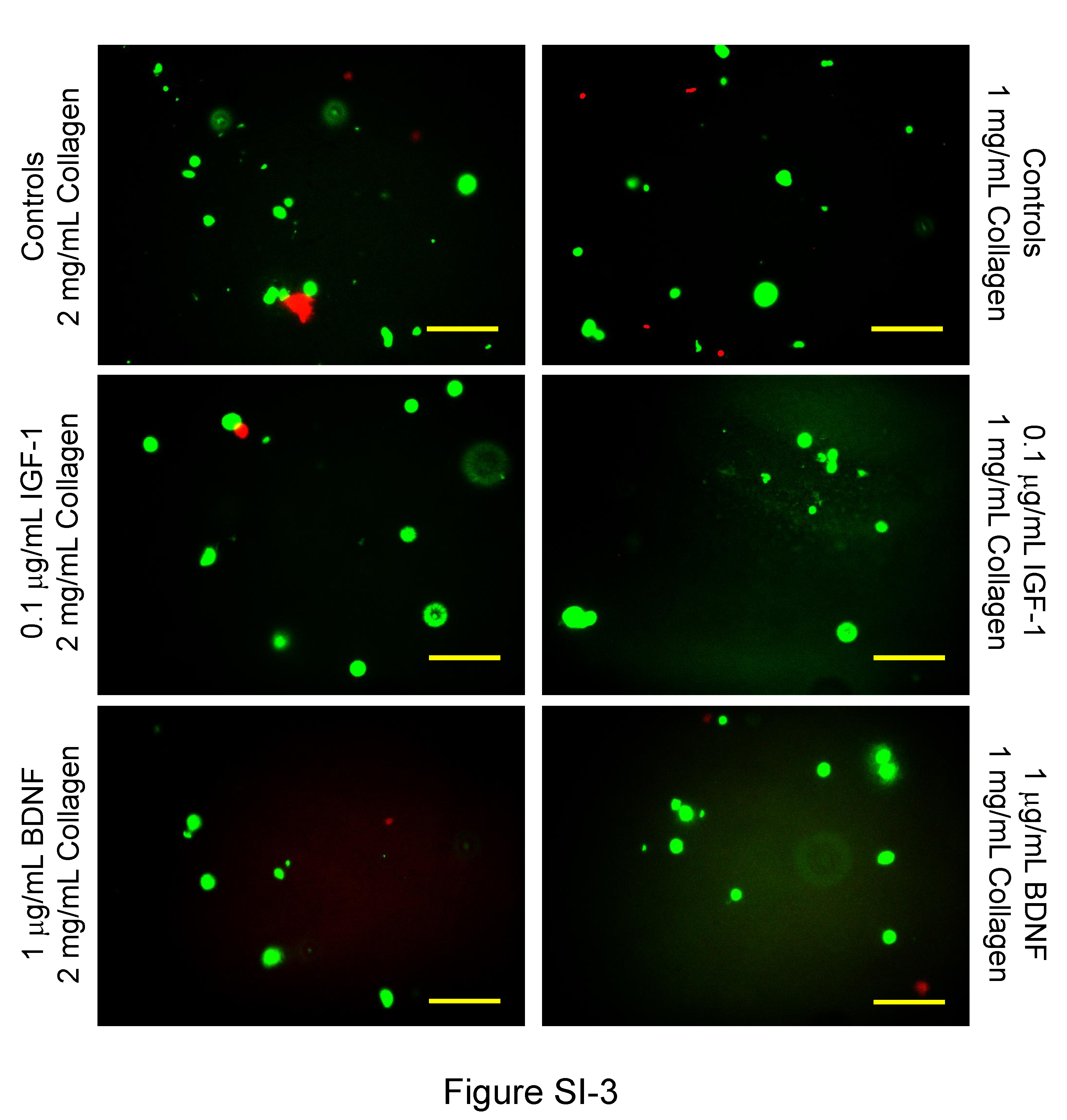

Supplement: Figure S3 — Cortical neuron survival within collagen gels under molecular gradients. Representative images of cell viability data from LIVE/DEAD assay, demonstrating high cortical neuron survival when cultured within 3D collagen scaffolds, and exposed to gradients of growth factor gradients for 48 h. Scale bar: 50 µm. (TIF) [file pone.0099640.s003.tif]

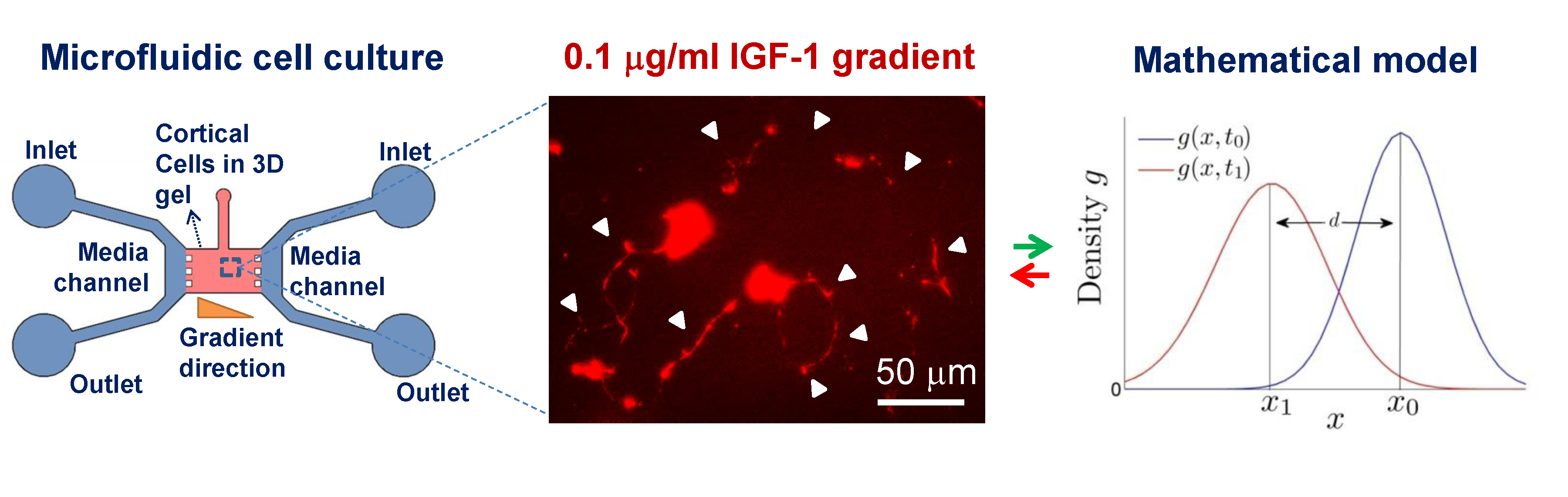

Supplement: Graphical Abstract S1 — (TIF) [file pone.0099640.s005.tif]
