# Supplementary material for: Synergistic Effects of 3D ECM and Chemogradients on Neurite Outgrowth and Guidance: A Simple Modeling and Microfluidic Framework
Source: PLoS One. 2014 Jun 10;9(6):e99640. doi: 10.1371/journal.pone.0099640 (PMC4051856; doi:10.1371/journal.pone.0099640)
Supplement: Methods S1 — Supporting data analysis. Statistical analysis of the data shown in Fig. 3D (Table A) and Fig. 3E (Table B). The model parameters and their respective values under various conditions tested in this study are also shown (Table C). (DOCX) [file pone.0099640.s004.docx]

1. Statistical analysis of the data shown in Fig. 3D

Here, * indicates significant differences in the percentage of neurites within a particular neurite length bin, for IGF-1 receiving cultures, compared to controls

|  | IGF-1 (μg/mL) | | |
| --- | --- | --- | --- |
|  | 1.0 | 0.1 | 0.01 |
| 0-10 | * | * | * |
| 11-21 |  | * | * |
| 21-30 | * | * |  |
| 31-40 |  | * | * |
| 41-50 | * | * | * |
| 51-60 | * |  | * |
| 61-70 | * |  |  |
| 71-80 | * |  |  |
| 81-90 | * | * |  |
| 91-100 | * |  |  |
| 101-110 | * | * |  |
| 111-120 | * |  |  |
| 121-130 | * | * |  |
| 131-140 | * |  |  |
| 141-150 | * | * |  |
| 151-160 |  |  |  |
| 161-170 | * |  |  |
| 171-180 | * |  |  |
| 181-190 |  |  |  |
| 191-200 | * |  |  |
| 201-210 |  |  |  |
| 211-220 | * |  |  |
| 221-230 |  |  |  |
| 231-240 |  |  |  |
| 241-250 | * |  |  |
| 251-260 | * |  |  |
| 261-270 |  |  |  |
| 271-280 |  |  |  |
| 281-290 |  |  |  |

1. Statistical analysis of the data shown in Fig. 3E

Here, * indicates significant differences in the percentage of neurites within a particular neurite length bin, for BDNF receiving cultures, compared to controls

|  | BDNF (μg/mL) | | |
| --- | --- | --- | --- |
|  | 1.0 | 0.1 | 0.01 |
| 0-10 | * | * | * |
| 11-21 | * | * | * |
| 21-30 |  | * | * |
| 31-40 | * |  |  |
| 41-50 | * |  |  |
| 51-60 | * |  |  |
| 61-70 |  |  |  |
| 71-80 |  |  |  |
| 81-90 | * |  |  |
| 91-100 | * |  |  |
| 101-110 | * |  |  |
| 111-120 | * |  |  |
| 121-130 | * |  |  |
| 131-140 |  |  |  |
| 141-150 | * |  |  |
| 151-160 |  |  |  |
| 161-170 | * |  |  |
| 171-180 |  |  |  |
| 181-190 | * |  |  |
| 191-200 | * |  |  |
| 201-210 |  |  |  |
| 211-220 | * |  |  |
| 221-230 | * |  |  |
| 231-240 |  |  |  |
| 241-250 |  |  |  |
| 251-260 | * |  |  |
| 261-270 |  |  |  |
| 271-280 |  |  |  |
| 281-290 | * |  |  |

1. Model parameters and their respective values under various conditions tested in this study.

| Scaffold | Growth factor | Model parameters | | |
| --- | --- | --- | --- | --- |
|  |  | D_c_ (μm^2^/s) | *k* (μm^2^mL/μg/s) | *m* (mL/μg) |
| 1 mg/mL collagen | IGF-1 | 17 | 10 | 10 |
|  | BDNF | 14 | 4.5 | 7 |
| 2 mg/mL collagen | IGF-1 | 8 | 9 | 15 |
|  | BDNF | 5.5 | 5.5 | 6 |
